# Challenges and Considerations in Selecting Animal Models for Evaluating a Live, Cold-Chain-Free, Dual-Use Vaccine (MyChol) for Diarrhoeal Diseases: A Comprehensive Review

**DOI:** 10.21315/mjms2024.31.5.4

**Published:** 2024-10-08

**Authors:** Hui Xian Tew, Parasuraman Subramani, Yean Yean Chan, Nik Zuraina Nik Mohd Noor, Prabhakaran Guruswamy

**Affiliations:** 1 Department of Biotechnology, Faculty of Applied Sciences, AIMST University, Kedah, Malaysia; 2 Department of Pharmacology, Faculty of Pharmacy, AIMST University, Kedah, Malaysia; 3 Department of Medical Microbiology and Parasitology, School of Medical Sciences, Universiti Sains Malaysia, Kelantan, Malaysia; 4 Centre of Excellence for Vaccine Development (CoEVD), AIMST University, Kedah, Malaysia

**Keywords:** dual-use vaccine, cholera, enterotoxigenic Escherichia coli (ETEC), New Zealand white rabbit, Sprague Dawley rat, BALB/c mice

## Abstract

Diarrhoeal diseases are the second leading cause of death for children under 5 years old in 69 low- and middle-income countries, with an annual economic burden of US$ 4 billion and over 525,000 lives lost. Cholera and enterotoxigenic *Escherichia coli* (ETEC) traveller’s diarrhoea are major diarrhoeal diseases caused by *Vibrio cholerae* (O1 and O139 serogroups) and ETEC, which have similar pathogeneses and can co-infect. There is no exclusive vaccine for ETEC, but cholera vaccines containing the cholera toxin B (CT-B) component offer short-term cross-protection. However, licensed oral cholera vaccines are expensive due to cold-chain supplies and the need for multiple doses. A cost-effective, dual-protection, live, cold-chain-free vaccine is, therefore, required for vaccination campaigns in low-resource settings, and MyChol – a prototype cold-chain-free live attenuated cholera vaccine, targeting *V. cholerae* O139 and ETEC H10407 – was developed in this context. The vaccine was evaluated in three animal models (Sprague Dawley [SD] rats, BALB/c mice and New Zealand white rabbits) for safety, colonisation capacity, reactogenicity and immunogenicity against challenge strains. In suckling mice, MyChol displayed high colonisation potential compared to unformulated VCUSM14P (the vaccine candidate) and wild-type O139. In the acute toxicity assessment, the SD rats with the highest MyChol dose (1 × 10^7^ colony-forming unit [CFU]/kg) demonstrated no adverse effects or mortality. Mice vaccinated with MyChol exhibited elevated antibody levels, including anti-CT, anti-heat-labile enterotoxin (LT), anti-CT-B and anti-LT-B. Anti-CT antibodies neutralised LT toxin in ETEC H10407 in challenge studies and cross-protected against ETEC H10407 in both mice and rabbits, preventing weight loss and diarrhoea. Ileal loop experiments in rabbits and BALB/c mice showed no reactogenicity. This review, based on our previous research, therefore provides valuable insights into improving the selection of animal models to advance preclinical evaluations of diarrhoeal vaccines.

## Introduction

Cholera and travellers’ diarrhoea are predominantly caused by two major versatile enteric bacterial pathogens: *Vibrio cholerae* and enterotoxigenic *Escherichia coli* (ETEC). Cholera is currently reported in 80 countries and around 40% of travellers to developing countries are prone to diarrhoeal disease. The pathogeneses of *V. cholerae* and ETEC are similar and characterised by toxin secretions; furthermore, cholera toxin (CT), produced by *V. cholerae* O1, and the heat-labile enterotoxin (LT) of ETEC share identical structural and functional features. Notably, most diarrhoeal patients are concurrently infected with both *V. cholerae* and ETEC, resulting in severe diarrhoea.

### Global Health Impact of Cholera and ETEC

Cholera poses a persistent threat to global health; it is a severe, acute diarrhoeal disease that can result in potentially fatal dehydration and is caused by the ingestion of food or water contaminated by the waterborne bacterium *V. cholerae* of serogroups O1 and O139 (1). Cholera outbreaks were reported in 23 countries in 2021, primarily in Africa and the Eastern Mediterranean, with the number increasing to 30 in 2022 (2). Similarly, ETEC diarrhoea is estimated to affect 220 million people worldwide annually, including 75 million cases in children under 5 years old of age, leading to between 18,700 and 42,000 deaths (3). Cholera and ETEC diarrhoea are diseases that predominantly affect the intestinal mucosa and cause complex – but different – immunological responses in humans and animals. *V. cholerae* colonises the mucosal surface of the human small intestine, multiplies and causes diarrhoea through the secretion of CT, and it is transmitted primarily through the ingestion of contaminated water or food (4). ETEC colonisation in the intestine is mediated by colonisation factor antigens and additional secondary adhesins.

### Current Vaccination Strategies and Challenges

The World Health Organization (WHO) Global Task Force on Cholera Control aims to reduce cholera deaths by 90% by 2030 through its Ending Cholera: A Global Roadmap to 2030 strategy and the WHO has also reaffirmed ETEC as a priority vaccine target (3). In endemic areas, large-scale oral cholera vaccination campaigns using the existing WHO-licensed oral cholera vaccines (OCVs) (Dukoral, Shanchol and Euvichol), which are based on whole-cell, killed *V. cholerae* O1 and O139 serogroups, are a proven strategy. Notably, killed vaccines provide short-term protection and require a booster dose, in contrast to single-dose live vaccine administration (5). Currently, there is no exclusive ETEC vaccine, although cholera vaccines based on the cholera toxin B (CT-B) component elicit a short-term cross-protection against ETEC infection. However, all these vaccines require a cold-chain supply (2 °C–8 °C), resulting in a 14%–20% increase in vaccination costs and repeated dosing costs, which poses a significant challenge. There is thus an urgent need for a cost-effective, dual-protection, cold-chain-free, live vaccine to manage diarrhoeal diseases in low-resource settings and enhance the outreach of global immunisation programmes.

### Development of the MyChol Vaccine

Live attenuated *V. cholerae* (VCUSM14P) was therefore constructed as a candidate vaccine against the O139 serogroup (patents EP1650315, US 7838016 and MY-142562A) to mimic natural infection and eliminate repeated dosing. Furthermore, a prototype of a live, oral, cold-chain-free, dual-protection vaccine formulation using the vaccine candidate was developed (patent MY-190074-A). This prototype, named MyChol, is a liquid suspension consisting of 5 × 10^7^ colony-forming unit [CFU]/mL of the live vaccine strain (VCUSM14P) and retains its potency at room temperature (25 °C ± 2 °C and relative humidity [RH] 60% ± 5%) for 180 days, in contrast to all existing WHO-licensed killed OCVs, which are dependent on cold-chain supply. MyChol was evaluated for its colonisation potential in a suckling infant mice model, for toxicity in a Sprague Dawley (SD) rat model, for reactogenicity in a rabbit ileal loop model, for protective efficacy in a reversible intestinal tie adult rabbit diarrhoea (RITARD) model and for immunogenicity in adult BALB/c mice and a New Zealand white (NZW) rabbit model (6, 7).

### Cross-Protection and Immunogenicity

The WHO pre-qualified cholera vaccine Dukoral contains inactivated *V. cholerae* and a recombinant CT-B subunit. This vaccine provides short-term protection against travellers’ diarrhoea because the CT produced by *V. cholerae* is similar to the heat-labile enterotoxin produced by ETEC. The prototype vaccine, MyChol, also contains two copies of CT-B and it overexpresses the B subunit of the CT. This may induce antibodies that can effectively cross-react with and neutralise the LT-B from ETEC. We therefore evaluated the cross-protection efficacy of MyChol against ETEC H10407 in an adult female BALB/c mice model (8). Based on our previous research, this review highlights the assessment of cholera vaccine efficacy through various animal models and its potential for cross-protection against ETEC infection, drawing from our 10 years of vaccine research, with implications for public health.

### Animal Models Used in the Development of Cholera and Traveller’s Diarrhoea Vaccine

Animal models continue to play a crucial role in vaccine development because there are no other methods currently available for assessing immunological responses and ensuring safety and efficacy before conducting human trials. Animal models are typically categorised into three groups: first, those used to evaluate immune responses; second, those that represent natural or surrogate disease models; and third, those designed for surgical or experimental interventions. Animal models are typically used to assess i) vaccine safety; ii) protection against challenge infection from the pathogen of interest; iii) dose and formulation of the vaccine (i.e. enhancement of the immune responses through adjuvants); iv) optimal route of delivery; v) onset, magnitude and duration of the immune response; vi) type of immunity and vii) correlates of protection (9).

One of the main challenges in developing and evaluating cholera vaccine candidates is that only humans are naturally susceptible to *V. cholerae* infection (10). This is because cholera immunity is complex and influenced by factors such as gut microbiota, adjuvants and mucosal, and adaptive immunity (11). Cholera research uses two main types of animal models – mammalian and non-mammalian – whose applications, strengths and limitations have been compared previously (12). Selecting suitable models is therefore crucial and must consider immune system variations, receptor expression and CT receptors in different animals. In this instance, the models selected should accurately represent two fundamental components of human cholera pathogenesis: toxin-coregulated pilus (TCP)-dependent colonisation of the small intestine and CT-dependent induction of diarrhoeal illness (13). Knowledge and choice of models plays a pivotal role in vaccine development, elucidating the protective mechanisms and advancing our understanding of vaccine-induced immunity. Nevertheless, researchers must comply with ethical standards, ensuring that the animals are treated with compassion.

Animal models vary significantly in terms of size, cost and the need for specialised infrastructure (14). Mice, rats and rabbits play crucial roles in evaluating and advancing cholera vaccines, while other animal models, such as chinchillas, guinea pigs, swine and monkeys are less susceptible to cholera and are generally not used for cholera vaccine research. Nevertheless, researchers have employed guinea pig models to investigate the mechanism of CT produced by *V. cholerae* (15, 16). Unlike the mice and rabbits commonly used in cholera research, guinea pigs exhibit lower susceptibility to *V. cholerae* but are susceptible to ETEC. ETEC also causes severe diarrhoea in neonatal, weaning and post-weaning piglets (17), and the cross-protective potential of a rice-based cholera vaccine (MucoRice-CTB) expressing CT-B against LT-ETEC infection has been evaluated in pigs (18, 19). Table 1 provides an overview of the different animal models used to evaluate cholera vaccines.

### Mice: Crucial Models for Cholera and Traveller’s Diarrhoea Vaccine Development

Mice are a cost-effective and practical choice for conducting research on cholera vaccines. They are easy to handle, with short lives and reproductive cycles. Their genetic and immunological backgrounds have been well studied along with their organ systems, which closely resemble those of humans, making them suitable for such studies (20). The variety of mouse models, such as BALB/c, C3H, C57BL/6, CBA, DBA/2, C57BL/10, AKR, A, 129, SJL and Swiss Webster, offer diverse research opportunities (21), and the ability to precisely introduce human disease-causing mutations into mice through modern sequencing and genomic engineering techniques has yielded more accurate and important data for disease research. Compared to using larger animal models, using mice in humane and controlled settings is typically regarded as more ethically acceptable (22).

The infant mouse cholera model allows for extensive research on the immunogenicity and colonisation of cholera vaccines (10). Infant mice display a high susceptibility to cholera and exhibit symptoms that closely resemble those observed in humans, and this model is thus especially relevant for simulating the manifestations of the disease (23). However, although the suckling mouse intestinal model is crucial for studying cholera’s pathogenesis and colonisation potential, its usefulness is limited to evaluating passive immunity due to the mice’s immature immune systems (24–27). Adult mice are ideal candidates for preclinical studies on both passive and acquired immunity, but there are discrepancies between mouse and human responses, which impact translatability (28, 29). Technical challenges, such as limited sample availability, further complicate the assessment of cholera vaccines in mouse models, as does the small size of mice compared to larger animal models, which poses a challenge in the development of surgical techniques (30).

### Evaluation of the Colonisation Potential, Immunogenicity and Protective Efficacy of MyChol against V. cholerae O139 and ETEC H10407 Challenges in Mice Models

#### Colonisation Potential of MyChol in Suckling Mice

The colonisation of *Vibrio* on the mucosal layer of the small intestine is crucial for inducing a protective immune response. To assess the colonisation potential of MyChol, a suckling mouse colonisation assay was conducted (7, 31). An inoculum containing from 10^3^ to 10^8^ *V. cholerae* cells is recommended for the effective infection of a human host (32), but a lower dosage of 10^5^ CFU of *V. cholerae* (El Tor strain C6706) has been found effective for both colonisation and biofilm formation in newborn mice (33). The MyChol live attenuated vaccine demonstrated similar results in a 3-day-old BALB/c mouse by successfully colonising at a lower dosage of 2.5 × 10^5^ CFU/50 μL. Compared to other VCUSM strains (VCUSM2, VCUSM14) (31, 34), the VCUSM14P strain used in MyChol exhibited a two-log higher recovery rate (7). This increased colonisation potential of the MyChol vaccine may be attributed to the mutation in ctxA, the deletion of the ace and zot genes and the presence of the hemA gene in the VCUSM14P strain.

#### Immunogenicity and Protection by MyChol against V. cholerae O139 and ETEC H10407 in Adult Mice

The challenge dose for *V. cholerae* O139 and ETEC H10407 was determined by evaluating different doses (1 × 10^7^ CFU/200 μL, 1 × 10^8^ CFU/200 μL and 1 × 10^9^ CFU/200 μL) in adult mice, as previously described (7, 35). The results showed that 1 × 10^9^ CFU/200 μL of ETEC H10407 was a 100% lethal dose (LD_100_) in BALB/c mice, and these results are similar to the previously reported 100% mortality of mice infected with 1 x 10^9^ CFU/200 μL of ETEC H10407. Among the mice that survived in the different groups, no significant body weight loss was observed and there were no signs of illness or diarrhoea before euthanasia after 14 days. These findings suggest that the adult mouse model is more susceptible to the ETEC H10407 strain than to *V. cholerae* O139. We therefore evaluated the cross-protection efficacy of MyChol against ETEC H10407 in adult female BALB/c mice (8). In this investigation, unimmunised and immunised BALB/c mice were challenged with an LD_100_ dose of 1 x 10^9^ CFU/200 μL of ETEC H10407 and observed for 14 days.

The mice that were immunised with MyChol demonstrated high tolerance after receiving both the initial and booster doses, and there were no adverse reactions or deaths observed for up to 28 days. The systemic IgG and IgA immune responses of the immunised animals increased when they were exposed to CT, which might be due to cross-reactivity with the LT toxin produced by ETEC. The immunised mice survived the ETEC H10407 challenge studies with a 100% survival rate and showed no signs of diarrhoea or weight loss, and a lower fluid accumulation ratio was recorded in their intestines after 24 h of ETEC challenge than in the unimmunised mice. No damage or loss of villi was observed in the immunised mice’s intestinal histopathological sections. These observations indicate that anti-LT and anti-LT-B antibodies were elicited in the immunised mice that hindered the LT and LT-B subunit of ETEC in binding to the GM1 ganglioside receptors on the epithelial cells of the intestine, preventing the endocytosis of LT into the cell. These observations are similar to those previously reported (36, 37).

The report on immunogenicity indicates a significant increase in serum IgG levels in response to CT (18-fold), CT-B (6-fold), LT (14-fold) and LT-B (4-fold) after the booster dose, as detected by enzyme-linked immunosorbent assay (ELISA). There was also a two-fold increase in serum IgA levels in response to CT, CT-B, LT and LT-B compared to baseline after the booster dose. After the challenge with ETEC H10407, there was an increase in anti-CT and anti-LT IgG/IgA levels, but no bactericidal antibodies against H10407 were found in the serum samples of the immunised mice. The lower IgG titres against LT/LT-B than against CT/CT-B might be due to specific antibodies induced by CT/CT-B that do not cross-react with LT/LT-B, despite an approximately 80% similarity. Various other cholera vaccines have also been evaluated for their anti-CT-B and anti-CT-A titres in adult mouse models, including Dukoral, Shanchol, CT-B (38), Euvichol (39), HaitiV (40) and Peru-15p CT-B (41).

### Rat Models: Assessing the Acute and Sub-Chronic Toxicity of MyChol

Similar to mice models, rat models are low cost, easily prepared and easily handled, with a well-characterised immune system. Rats are also larger than mice, which makes surgical procedures and collecting samples less challenging (29). Unfortunately, rat models are not typically used in research to evaluate the immunogenicity and protective efficacy of cholera vaccines due to their lack of natural susceptibility to the disease and their differences in physiology from humans. Rats exposed to the CT produced by *V. cholerae* exhibit a hypersecretory state and an increase in intestinal fluid content. The cecum serves a reservoir function in the rat during periods of small intestinal hypersecretion caused by CT (42); this would make secretory diarrhoea in rats extremely difficult to elicit, which appears to be the reason for the inability to properly develop an acceptable model of diarrhoea in intact rats. Additionally, the rat colon is not like the human colon in that it lacks a substantial fluid absorptive reserve. However, the adult SD rat has been considered a relevant biological model for evaluating toxicity induced by vaccine products against cholera after repeated doses (43, 44). An oral tablet with an inactivated whole-cell cholera vaccine with the *V. cholerae* C7258 strain was administered to SD rats in three fixed doses for toxicological evaluation (43). Another study assessed the toxicity of three doses of an inactivated oral cholera vaccine consisting of five *V. cholerae* O1 and O139 strains administered every two weeks to SD rats (45).

We evaluated the safety of MyChol, a live attenuated vaccine candidate of the VCUSM14P strain, in the SD rat model using single and repeated doses (30 doses for 30 days) to assess its suitability for clinical use (6). Single-dose toxicity (acute toxicity) experiments with MyChol were conducted to determine the doses that SD rats could tolerate for long-term repeat-dose (sub-chronic) investigations. The SD rats that were administered MyChol at the highest dose (1 × 10^7^ CFU/kg) showed no adverse effects or mortality in the acute toxicity study. In the subsequent repeated toxicity study, three different concentrations of the vaccine (1.25 × 10^6^ CFU/kg, 2.5 × 10^6^ CFU/kg and 5.0 × 10^6^ CFU/kg) were tested (a single dose per day for 30 days) to determine the no observed adverse effect level (NOAEL) according to Organisation for Economic Cooperation and Development (OECD) guidelines. The NOAEL dose was determined to be 1.25 × 10^6^ CFU/kg (6). No significant differences were observed in biochemical and haematological analyses for any the experimental SD rats, and mild to severe histopathological changes were observed in the organs, which can be attributed to the 30 doses of vaccine given in daily succession without an interval. This investigation is the first to report the repeated toxicity of a live attenuated, cold-chain-free oral cholera vaccine at three different concentrations administered daily for 30 days in SD rats.

### Rabbit Models for Evaluating the Reactogenicity, Immunogenicity and Protective Efficacy of MyChol

The rabbit model is another animal model vulnerable to cholera infection and to developing clinical signs comparable to cholera cases in humans (46). Rabbits are bigger than mice and have a more human-like gastrointestinal system, making it possible to examine the disease’s development in greater detail and providing information that may be more applicable to human infection. The NZW rabbit is the breed most frequently used in research – more frequent than the California and Dutch-belted rabbit breeds (20). Rabbits are used as a model for the production of polyclonal antibodies for use in immunology research due to their relatively large blood volumes compared to rodents (47). Oral administration of *V. cholerae* to adult animals typically does not result in cholera-like symptoms; to overcome this obstacle, surgical techniques have been developed that directly inoculate *V. cholerae* into the small intestine, most commonly the rabbit ileal loop model and the RITARD model (48, 49).

#### Evaluation of the Reactogenicity of MyChol in the Rabbit Ileal Loop Model

The safety of the live attenuated vaccine was assessed for reactogenicity using the fluid accumulation ratio (FAR) in a rabbit ileal loop model. Ligated rabbit ileal loop assays were performed in both unimmunised and immunised rabbits, as described in (7, 31, 34). A FAR of greater than 1.0 indicates strong toxigenicity of CT, while a FAR of less than 0.2 indicates no reactogenicity (50, 51). Our investigation found that MyChol did not exhibit detectable diarrhoeagenic activity (FAR < 0.2), even at an inoculation dose of 10^4^ CFU/mL– 10^6^ CFU/mL. There were no signs of haemorrhage or reactogenicity observed in the rabbits immunised against wild-type *V. cholerae* strain O139 and ETEC strain H10407 compared to the unimmunised rabbits. In the rabbits immunised with MyChol, a FAR of less than 0.2 was observed in the loops injected with 1 × 10^5^ CFU/mL of wild-type O139 or ETEC H10407 and a ~50% reduction in FAR was recorded in the loops injected with 1 × 10^6^ CFU/mL. These findings suggest that the MyChol-immunised rabbits developed anti-CT antibodies to neutralise the LT toxin produced by the H10407 strain in the intestines, thereby reducing the FAR. While ligated rabbit loops can be used to study intestinal secretions triggered by *V. cholerae*, this model requires complex surgery and bypasses the normal route of infection, as mentioned in (48, 52). It is thus not suitable for studying intestinal colonisation factors.

#### Determination of the Immunogenicity Profile of MyChol in Rabbits

Antitoxin and bactericidal antibodies are indicators of the protective efficacy of a vaccine against enteric infections (53, 54). In our study, adult NZW rabbits were immunised with MyChol to assess its safety and immunogenicity (7). None of the immunised rabbits showed any mortality, diarrhoea symptoms, clinical signs or body weight loss after the first booster dose. The immune response of the immunised rabbits was determined by measuring the anti-CT IgG, anti-CT IgA, anti-LT IgG, anti-LT IgA, anti-CT-B IgG, anti-CT-B IgA, anti-LT-B IgG, anti-LT-B IgA and bactericidal antibodies. The ELISA results show a significant increase in serum IgG titres to CT (50-fold), CT-B (45-fold), LT (22-fold) and LT-B (19-fold) after the booster dose (Table 2). An increase of 9 to 14 times over the baseline was observed in serum IgA titres to CT, CT-B, LT and LT-B after the booster dose. In all the serum samples from immunised rabbits, there were no bactericidal antibodies against H10407 but there were against *V. cholerae* O139 (Table 3). These immunological results indicate that the immunised rabbits successfully developed systemic (IgG) and mucosal immunity (IgA) against CT, which cross-reacted with the LT toxin produced by ETEC. The anti-LT/LT-B IgG titres were also lower than the anti-CT/CT-B titres. These immunogenic findings are complementary to the findings regarding the cross-protection of MyChol against ETEC in mice described earlier (8). The anti-CT antibodies showed a similar trend to those in rabbits immunised with *Vibrio cholerae* ghost (VCG) candidate (55), IEM108 (56), VA 1.4 (54) and Peru-15 (57). The anti-LT antibody results are compared to the rabbits immunised with Peru-15p CT-B, a live cholera vaccine consisting of a plasmid carrying the gene for the nontoxic B subunit of cholera toxin (ctxB), which elicits higher titres of anti-LT IgG and IgA (41).

#### Protective Efficacy in the Reversible Intestinal Tie Adult Rabbit Diarrhoea (RITARD) Model

A RITARD assay was conducted to validate the results of the rabbit ileal loop assay and determine the efficacy of the vaccine against challenges with *V. cholerae* O139 and ETEC H10407. Unimmunised rabbits challenged with 1 × 10^9^ CFU/mL of wild-type O139 showed a 100% mortality rate within 24 h, while immunised rabbits showed no mortality and exhibited no diarrhoea symptoms for up to 5 days. These results are in agreement with previous studies on VCG vaccine candidates (55), purified outer membrane vesicles (OMVs) (58) and live attenuated cholera vaccine VA1.4 (54). Unimmunised rabbits challenged with 1 × 10^9^ CFU/mL and 1 × 10^10^ CFU/mL of ETEC H10407 did not exhibit any mortality. Our results corroborate those of (59, 60), which used the RITARD model to challenge rabbits with ETEC H10407 at doses of 1 × 10^10^ and 1 × 10^11^ cells. The findings suggest that rabbits are less susceptible to ETEC strains than to *V. cholerae* strains in RITARD models, which may be due to ETEC’s ability to produce toxins and adhere to the intestines, causing symptoms of diarrhoea, and to the animals’ increased resistance to ETEC as they age. The use of the RITARD model in ETEC studies is less common due to its invasive surgical nature and the stress it imposes on the animals (61). Table 4 provides a summary of the key findings from all the case studies.

### Challenges and Considerations in the Selection of Appropriate Animal Models

Selecting appropriate animal models for research on cholera vaccines is a complex task because it requires models that closely resemble human physiology in terms of genetics, anatomy and metabolism, which is crucial to ensure the validity of the findings (62). However, species-specific variations in drug metabolism, toxicity and treatment responses complicate this process, and identifying accurate biomarkers reflecting human responses is crucial for research relevance (63).

#### Mice Models

We employed a mice model to evaluate the colonisation potential, immunogenicity and protective efficacy of the cholera vaccine MyChol when challenged by *V. cholerae* O139 and ETEC H10407. Adult BALB/c mice were used to assess vaccine immunogenicity and protective efficacy. This study unequivocally showed that ETEC H10407 is more lethal than *V. cholerae* O139 in the BALB/c mice model, underscoring the value of assessing vaccine efficacy within this specific context. During this investigation, we encountered challenges in obtaining adequate blood samples from the test animals, primarily due to limited blood volume (less than 200 μL) for the immunological, biochemical and haematological analyses. Moreover, due to the small size of the mice, intricate surgical techniques were required for precise organ collection during the histopathological analysis.

#### Rat Models

We conducted acute and sub-chronic toxicity studies using adult SD rats to evaluate the safety of MyChol. However, we observed that SD rats, despite being susceptible to both the *V. cholerae* and ETEC strains, are not suitable for assessing the vaccine’s immunogenicity and protective efficacy and do not manifest the diarrhoea symptoms induced by either strain.

#### Rabbit Models

Rabbit models were used to evaluate the reactogenicity, immunogenicity and protective efficacy of MyChol. The main limitation of the rabbit model is its higher cost, which can lead to a smaller research sample size, alongside ethical concerns than in mice and rat models. Since oral administration of *V. cholerae* and ETEC to adult rabbits usually does not induce diarrhoea symptoms, we conducted rabbit ileal loop and RITARD procedures to simulate cholera and ETEC infections. Specific training is essential before conducting these surgical experiments because one of the challenges during the procedures was the administration of anaesthesia to alleviate pain and discomfort in the rabbits. In summary, MyChol was found to be non-reactogenic and immunogenic in vivo and to protect animals from lethal wild-type *V. cholerae* O139 and ETEC H10407 challenge in BALB/c mice and NZW rabbits. The major considerations and challenges involved in assessing MyChol using the three animal models are summarised in Table 5.

## Conclusion

This comprehensive review, based on extensive studies, emphasises the critical importance of selecting accurate animal models for precise evaluation in cholera and ETEC vaccine development. Our results demonstrate that MyChol is effective against *V. cholerae* O139 and ETEC challenges in mouse, rat and rabbit models. Infant BALB/c mice are particularly valuable for assessing colonisation, while adult BALB/c mice and NZW rabbits are ideal for testing reactogenicity, immunogenicity and protective efficacy, and the SD rat model is critical for assessing toxicity. This careful selection of models not only enhances our understanding of vaccine performance but also shows a way for more refined preclinical trials and future advances in the development of cholera and ETEC vaccines for diarrhoeal diseases.

## Figures and Tables

**Table 1:** Summary of animal models used to evaluate the cholera vaccines

Animal models	Areas of research	Advantages	Disadvantages
**Infant mice**	Colonisation potential	Cost-effective Easily handling Easily accessible Short reproductive cycle Immune system of mice resembles humans and is well characterised	Small animal Difficult to collect and analyse the samples Immature immune system No disease symptoms
**Adult mice**	Reactogenicity Immunogenicity Protective efficacy	Cost-effective Easily handling Easily accessible Short reproductive cycle Genetic engineering accessible for study Immune system of mice resembles humans and is well characterised Beneficial for early vaccine screening and basic research	Small animal Difficult to collect and analyse the samples Require antibiotics No disease symptoms
**Rat**	Safety Toxicity	Cost-effective Easily handling Easily accessible Short reproductive cycle	Not susceptible to *Vibrio cholerae*
**Infant rabbit**	Colonisation potential Immunogenicity Protective efficacy	Cost-effective Susceptible to cholera toxin	Immature immune system No disease symptoms
**Adult rabbit**	Reactogenicity Immunogenicity Protective efficacy	Cost-effective Susceptible to cholera toxin Can perform surgical	Require antibiotics Require surgical technician

**Table 2:** Geometric mean titre (GMT) of anti-CT/CT-B IgG and anti-LT/LT-B IgA elicited in rabbits vaccinated with MyChol

Immune response *n* = 5	GMT (range) on the day of immunisation		

	Pre-immunisation	14 days After the first dose	28 days After booster dose
Anti-CT IgG	10.00 (10)	376.41 (160–1280)	590.02 (320–2560)
Anti-CT IgA	10.00 (10)	145.73 (40–320)	149.49 (80–320)
Anti-LT IgG	10.00 (10)	219.92 (80–640)	232.58 (80–640)
Anti-LT IgA	10.00 (10)	63.86 (20–160)	104.48 (40–320)
Anti-CT-B IgG	10.00 (10)	268.06 (80–1280)	462.71 (160–2560)
Anti-CT-B IgA	10.00 (10)	81.46 (40–320)	110.45 (40–320)
Anti-LT-B IgG	10.00 (10)	144.72 (40–320)	202.85 (160–320)
Anti-LT-B IgA	10.00 (10)	54.35 (20–320)	97.70 (20–320)

Notes: CT = cholera toxin; LT = heat-labile enterotoxin; CT-B = cholera toxin B subunit; LT-B = heat-labile B subunit; IgG = Immunoglobulin G; Ig = Immunoglobulin A

**Table 3:** Bactericidal antibodies against *V. cholerae* O139 and ETEC H10407 elicited in rabbits immunised with MyChol

Bactericidal antibodies *n* = 5	Day of immunisation		

	Pre-immunisation	14 days After the first dose	28 days After booster dose
*V. cholerae* O139	Absent	Present	Present
ETEC H10407	Absent	Absent	Absent

**Table 4:** Comparison of the overall findings of using different animal models for the evaluation of cholera vaccine MyChol

	Mice (BALB/c)		Rat (Sprague Dawley)	Rabbit (New Zealand White)

	Infant	Adult	Adult	Adult
Colonisation potential	VCUSM14P is a good coloniser	Reduced the ETEC H10407 colonise in the small intestine of immunised mice	X	X
Reactogenicity	X	Reduced the fluid accumulation caused by an enterotoxin of ETEC H10407 in the small intestine of immunised mice	X	MyChol do not cause any reactogenicity effect rabbit ileal loop model Significantly reduced the fluid accumulation (~50%) caused by the enterotoxin of *V. cholerae* WT O139 and ETEC H10407 in the rabbit ileal loop model
Immunogenicity	X	Elicited anti-CT and anti-LT antibodies No bactericidal antibodies against H10407 were found but against *V. cholerae* WT O139	X	Elicited anti-CT and anti-LT antibodies No bactericidal antibodies against H10407 were found but against *V. cholerae* WT O139
Protective efficacy	X	Cross-protected the immunised mice from ETEC H10407 challenge studies	X	Protected the immunised rabbits from *V. cholerae* WT O139 challenge studies by performing the RITARD model
Toxicity	X	X	No adverse effect and lethality was found in the acute toxicity study with a single dose of vaccine up to 10 x 10^6^ CFU/kg	X

Note: X = not evaluated

**Table 5:** Consideration and challenges using three different animal models for evaluation of MyChol

Consideration and challenges	Animal models		

	BALB/c mice model	Sprague Dawley rat model	New Zealand White rabbit model
Sample size	Min six animals per group	Min six animals per group	Min five animals per group
Gender	Female and male	Female	Male
Cost	Low	Medium	High
Blood sample collection	< 200 μL (orbital sinus)	1 mL (orbital sinus)	5 mL (marginal ear vein)
Organ sample collection	Difficult	Medium	Medium
Ethical concerns	Low	Low	High
Surgical procedure	Difficult	Moderate	Moderate
Susceptibility to *V. cholerae*	Low	No	High (in RITARD model)
Susceptibility to enterotoxigenic *E. coli* (ETEC)	High	No	Low (in RITARD model)

Use for evaluation of vaccine in different aspects: Colonisation efficacy Safety Reactogenicity Immunogenicity Protective efficacy	Infant mice used to evaluate the colonisation potential of the vaccine Adult mice used to evaluate the safety, immunogenicity and protective efficacy of the vaccine	Adult rats used to evaluate the toxicity of the vaccine. (acute and sub-chronic)	Adult rabbits used to evaluate the reactogenicity, immunogenicity and protection efficacy of the vaccine. (Rabbit ileal loop and RITARD assay
